# Artifact Removal using Improved GoogLeNet for Sparse-view CT Reconstruction

**DOI:** 10.1038/s41598-018-25153-w

**Published:** 2018-04-30

**Authors:** Shipeng Xie, Xinyu Zheng, Yang Chen, Lizhe Xie, Jin Liu, Yudong Zhang, Jingjie Yan, Hu Zhu, Yining Hu

**Affiliations:** 1 Nanjing University of Posts and Telecommunications, College of Telecommunications & Information Engineering, Nanjing, Jiangsu 210003 China; 2 LIST, Key Laboratory of Computer Network and Information Integration, Ministry of Education, Southeast University, Nanjing, 210096 China; 3 International Joint Research Laboratory of Information Display and Visualization, Southeast University, Ministry of Education, Nanjing, 210096 China; 4 Department of Informatics, University of Leicester, Leicester, LE1 7RH UK; 5 Jiangsu Key Laboratory of Oral Diseases, Nanjing medical university, Nanjing, 210029 China

## Abstract

Sparse-view Reconstruction can be used to provide accelerated low dose CT imaging with both accelerated scan and reduced projection/back-projection calculation. Despite the rapid developments, image noise and artifacts still remain a major issue in the low dose protocol. In this paper, a deep learning based method named Improved GoogLeNet is proposed to remove streak artifacts due to projection missing in sparse-view CT reconstruction. Residual learning is used in GoogLeNet to study the artifacts of sparse-view CT reconstruction, and then subtracts the artifacts obtained by learning from the sparse reconstructed images, finally recovers a clear correction image. The intensity of reconstruction using the proposed method is very close to the full-view projective reconstructed image. The results indicate that the proposed method is practical and effective for reducing the artifacts and preserving the quality of the reconstructed image.

## Introduction

X-ray Computed Tomography (CT) techniques have been widely utilized in clinical for diagnosis and intervention, including imaging, image-guided needle biopsy, image-guided intervention, and radiotherapy with noticeable benefits. However, with the broadened application of CT in clinical scenarios, the radiation risk issue is receiving more and more attention. As a result, the demand of radiation dose reduction is becoming more and more intense under the principle of ALARA (as low as reasonably achievable). Despite the rapid developments, image noise and artifacts still remains a major issue in the low dose protocol. Balancing image quality and x-ray dose level has become a well-known trade-off problem. Basically, low dose CT can be achieved reducing the tube currents (or voltage) or projection numbers. The approach of tube current (or voltage) reduction sacrifices image quality for dose reduction. Projection number reduction can be realized by applying sparse-view protocol for a given scanning trajectory. CT reconstruction with this approach is termed sparse-view CT reconstruction in this study. Compared to tube current or voltage reduction, sparse-view CT reconstruction does not suffer from the increased noise in projections and has the additional benefit of accelerated scan and projection/back projection calculation. Nevertheless, sparse-view CT reconstruction suffers from image quality deterioration caused by the increased streaking artifacts due to missing projections.

A great effort has been devoted to improve sparse-view CT reconstruction in the past twenty years. Specifically, by accommodating measurement statistics, modeling data acquisition geometry, and enforcing physical constraints, regularized iterative reconstruction algorithms often produce superior image quality with highly noisy measurements, and hence having become increasingly popular. In 2006, Donoho proposed the concept of compressed sensing (CS) and proved that sparse signals or piecewise images could be satisfactorily reconstructed from far less sampling data than the requirement of the Nyquist sampling theorem.

Base on the CS theory, a state of art solution, which is called as adaptive steepest descent projection onto convex sets (ASD-POCS) method^1^, was invented by Sidky *et al*. by minimizing the total variation (TV) of the desired image for CT image reconstruction from sparse projection views. Recently, a more general term of TV minimisation, called adaptive-weighted total variation (AwTV) model^2^, was proposed to improve the preservation of edge details by bringing the local information into the above conventional TV model.

To eliminating the patchy artifacts and preserving subtle structures, Liu *et al*. proposed a total variation-stokes-projection onto convex sets (TVS-POCS) method^3^ for the purpose of recovering possible missing information in the sparse-view data situation.

Although these TV-based algorithms are successful in a number of cases, the power of the TV minimization constraint is still limited. Besides the TV-based method and its general case, a prior image-constrained compressed sensing (PICCS) method^4^ and patch based nonlocal means (NLM)^5^, tight wavelet frames, feature dictionary learning^6,7^, low rank methods and so on, were introduced to further reduce the number of required projection views by incorporating prior images or patch information to the CS theory.

Compared to TV based method, such approaches have the potential of achieving better performance in representing patch-wise structure features and leading to better CT image quality.

Recently, Deep Learning techniques have recently been considered to improve CT reconstruction quality. H. C. Burger at al proposes a Multi-Layer Perceptron (MLP) machine based method to learn the mapping from the noisy images to the corresponding noise-free images and obtain an impressive performance in image restoration^8–10^. However, the application of MLP with fully connected layers is often limited by the requirement of fixed input/output size and the weight parameter explosion in network training. J.K. Batenburg and D. Pelt proposed introduce a new CT reconstruction method that improves the filtered back projection method by using a custom data-dependent filter that minimizes the projection error of the resulting reconstruction^11^. Li *et al*.^12^ proposed a dictionary-based sinogram completion method to inpaint the missing sinogram data by applying K-SVD algorithm^13^, with database composed by the patches from simulated CT sinogram. Chen *et al*.^14,15^ proposed a new sinogram restoration approach (Sinogram Discriminative Feature Representation) to improve projection data inconsistency. Lee *et al*.^16^ applied convolution neural network (CNN) to interpolate missing data of sinogram for sparse-view CT, by combining with residual learning for better convergence and patch-wisely training the network to avoid memory problem.

Würf *et al*.^17^ and Ma *et al*.^18^ mapped FBP algorithm into a deep CNN architecture that allowed a data-driven approach for joint optimization of correction steps in projection domain and image domain. Cheng *et al*.^19^ simulated the iterative process using a DL based leapfrogging strategy. The method was applied to speed up a penalized likelihood PET image reconstruction algorithm, block sequential regularized expectation maximization. In^20^, a residual convolutional network architecture was designed to build the relationship between the wavelet coefficients of low-dose and high-dose CT images. Han *et al*.^21^ proposed a U-net structured architecture with residual learning to predict the artifacts in sparse-angle reconstructed CT image. A residual learning of deep CNN method was reported in^22^ for image de-noising. Jin *et al*.^23^ proposed a deep convolutional network (FBPConvNet) that combines FBP with a multi-resolution CNN based on Unet^24^ and residual learning^25^.

## Results

### Experimental Design

In this section, the well-known filtered back-projection (FBP) reconstruction method is performed and the residual learning is used to remove artifacts generated during sparse-view reconstruction.

There are 16000 slices of images for each types of the training data, and 1600 slices of images for each types of the test data. For the training data set, we use the FBP reconstruction using 60 and 120 projection views (full scan) as input x and the difference between the full-view (720 views) reconstruction and the sparse view reconstructions are used as label *f*. The architectural parameters are described in Table 1.

**Table 1:** Incarnation of the architecture.

Type	Patch size/stride
convolution	3 × 3/1
ReLu	
convolution	64 filters of size 3 × 3/1
BN + ReLu	
Inception …	8 same inception model layers
convolution	3 × 3 × 64/1

The acquisition parameters of the experimental scans are defined in Table 2. To evaluate the imaging performance of the proposed method under realistic conditions, a set of clinical data and images were used. The image dataset contains 2000 full dose 512*512 CT images. The reference slices were generated using FBP method from 720 projection views. We calculate synthetic projections using fan-beam geometry and projections data were down-sampled to 60 and 120 views to simulate the few-view geometry.

**Table 2:** acquisition parameters of the experimental scans.

Data	Thorax
Distance Source to Detector	988.00 mm
Distance Source to Patient	560.00 mm
Scanner mode	Helical
Tube voltage	100 KVp
Tube current	240 mA
Detector size	313.89 mm × 313.89 mm
reconstruction	512 × 512 × 640
Volume size	0.61 × 0.61 × 0.3125 mm^3^

### CT data analysis

**Figure 1:**
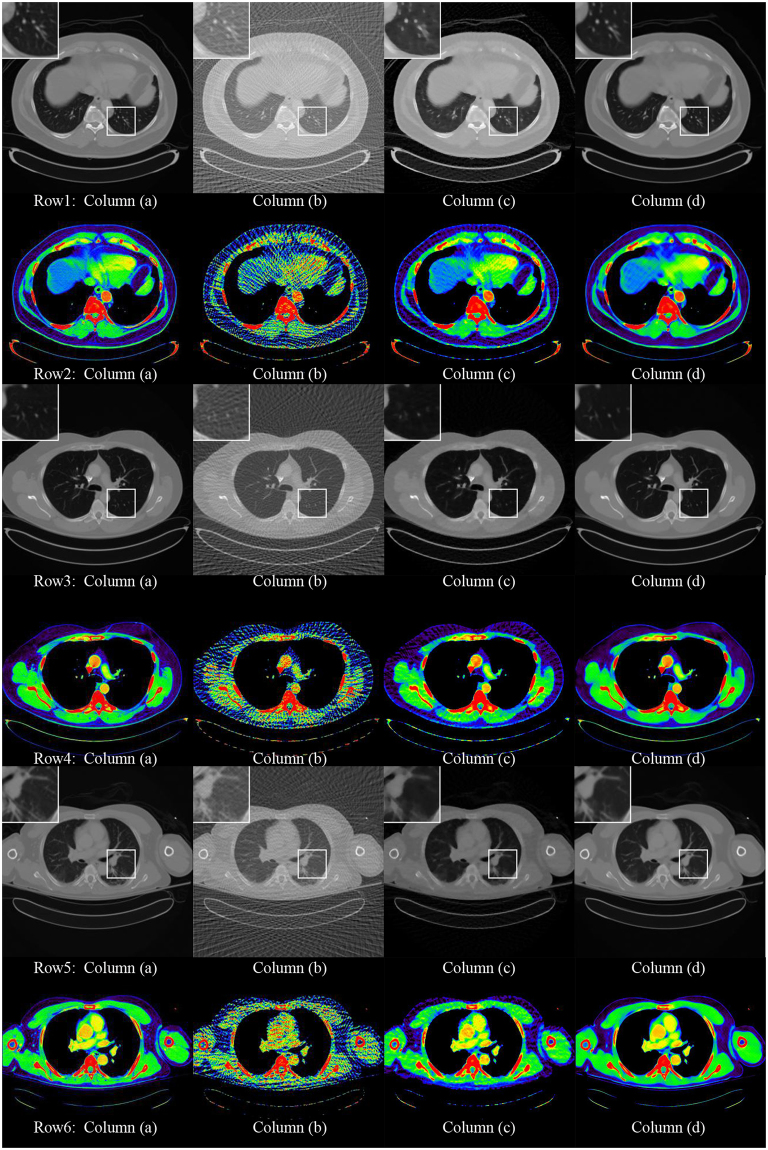
The 512*512 CT images reconstructed by 120 projection views. Column (**a**) shows the reconstruction by the FBP method from the full projection views. Column (**b**) shows the reconstruction by the FBP method from the sparse projection views. Column (**c**) shows the reconstruction by the ADS-POCS method from the sparse projection views. Column (**d**) shows the reconstruction by the proposed method. All images display in the same window at row 1, 3 and 5. In order to compare the CT value with different reconstruction method, we use 2D atlas to display. The result is shown in row 2, 4 and 6.

**Figure 2:**
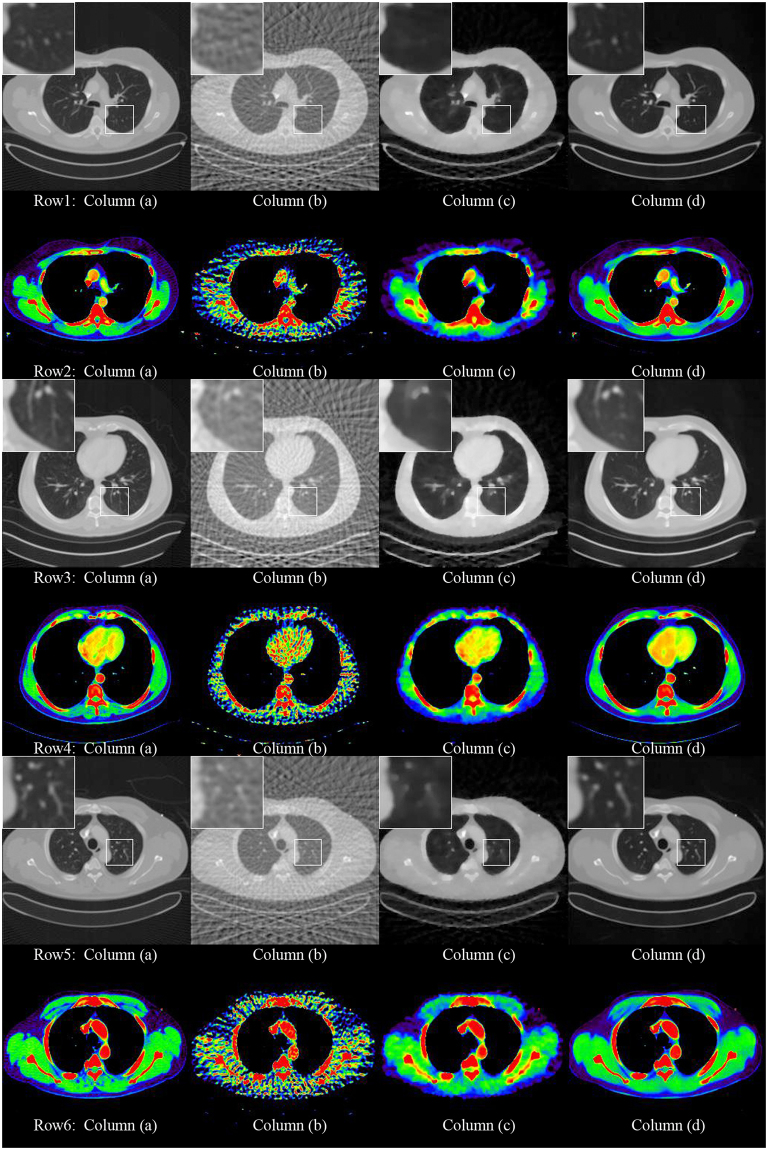
The 512*512 CT images reconstructed by 60 projection views. Column (**a**) shows the reconstruction by the FBP method from the full projection views. Column (**b**) shows the reconstruction by the FBP method from the sparse projection views. Column (**c**) shows the reconstruction by the ADS-POCS method from the sparse projection views. Column (**d**) shows the reconstruction by the proposed method. All images display in the same window at row 1, 3 and 5. In order to compare the CT value with different reconstruction method, we use 2D atlas to display. The results are shown in row 2, 4 and 6.

In this section, sparse view CT reconstruction input images are generated using FBP from 120 (3° angle increment for the tube), and 60 (6° angle increment for the tube) projection views, respectively. The raw data are exported from clinical routine CT examinations. Artifact-free original images are generated by FBP which uses all 720 projection views. The data of the 720 projections are used as full-view projection. We assumed the reconstruction from the full-view data using FBP algorithm as our gold standard image. As we all know, after reducing the number of projections, there is a large number of stripe artifacts in the reconstructed image. As shown in Figs 1 and 2, the column (a) shows the full-view projection. The column (b) shows the sparse reconstruction of the 120-views and 60-views using FBP. The column (c) shows ADS-POCS method [1] of the 120-views and 60-views and the column (d) is our method.

Peak signal-to-noise ratio (PSNR) and stands for structural similarity (SSIM) index is related to evaluate quality of the reconstructed image. PSNR is based on the error between corresponding pixels. The unit of PSNR is dB, the larger value means smaller distortion. SSMI measures image similarity from three aspects of brightness, contrast and structure respectively. The range of SSIM is 0 to 1. Similarly, the larger value represents smaller image distortion. The average PSNR and SSIM between the results and full projection reconstructed images are calculated and shown in Table 3. As shown in Table 3, the PSNR values and the SSIM values have a very pleasant value. The stored data is 12 bits.

**Table 3:** Average values of PSNR and SSIM between proposed method and full-view FBP reconstruction for 512*512 CT images.

512*512	120-view PSNR	60-view PSNR	120-view SSIM	60-view SSIM
FBP	29.32	24.97	0.5430	0.3504
ADS-POCS	40.66	35.12	0.9557	0.8941
Our method	**46.8**	**42.02**	**0.9880**	**0.9642**

### Time consumption

This article uses MatConvNet toolkit in the training process, the running environment is MATLAB 2017a. In the train stage, the processor is Intel Xeon E5-2650, the memory is 128 GB. We use one GeForce GTX 1080TI GPU video card for train. In this configuration environment, it takes about 72 hours to train samples over the network for one type of data. In the test stage, the processor is Intel (R) Core (TM) i7 CPU@ 2.2 GHz, the memory is 16GB. In this stage configuration environment, the time consumption is show in Table 4.

**Table 4:** Time consumption of different methods.

	120-view (unit: s)	60-view (unit: s)
FBP	0.21	0.10
ADS-POCS	16.3	14.6
Our method	**4.1**	**4.1**

## Discussion

**Figure 3:**
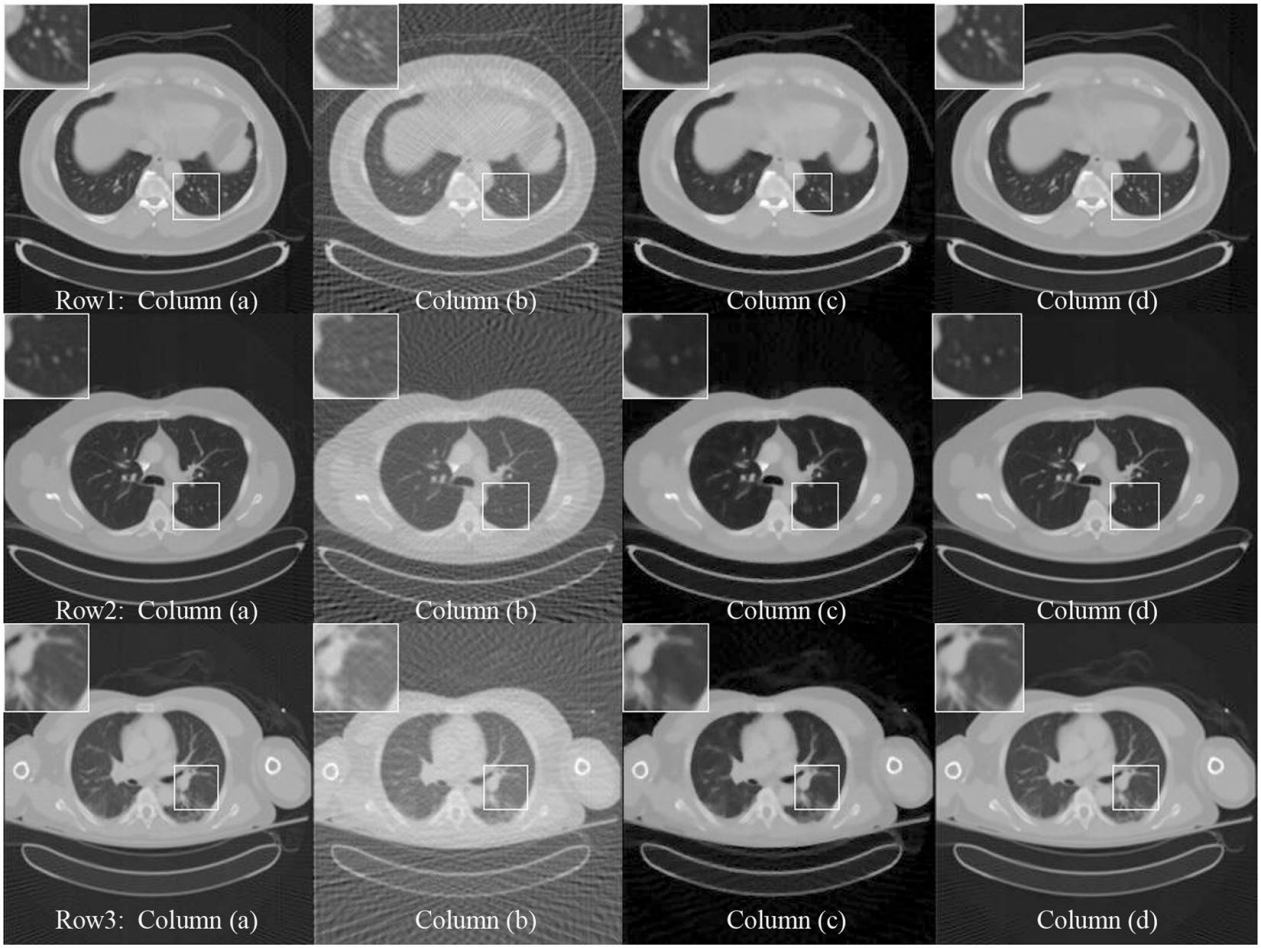
The 256*256 CT images reconstructed by 120 projection views. Column (**a**) shows the reconstruction by the FBP method from the full projection views. Column (**b**) shows the reconstruction by the FBP method from the sparse projection views. Column (**c**) shows the reconstruction by the ADS-POCS method from the sparse projection views. Column (**d**) shows the reconstruction by the proposed method.

As shown in the zoomed ROI images at the top of Figs 1, 2 and 3, the proposed method displays a very good image quality. Compared to the gold standard image, the FBP result suffered from the artifacts in a high degree. The algorithms can greatly remove the artifacts than the ADS-POCS. The artifacts of reconstructed images after different sparse-view training is greatly removed by the proposed method.

As shown in Fig. 3, 256*256 CT images are used to remove the artifacts. The PSNR and SSIM index can be better by using the lower resolution in the experiments. The average PSNR and SSIM between the results and full projection reconstructed images are calculated and shown in Table 5. As shown in Table 5, the PSNR values and the SSIM values have a very pleasant value.

**Table 5:** Average values of PSNR and SSIM between proposed method and full-view FBP reconstruction for 256*256 CT images.

256*256	120-view PSNR	60-view PSNR	120-view SSIM	60-view SSIM
FBP	29.9	26.39	0.6890	0.4383
ADS-POCS	41.76	36.01	0.9745	0.9174
Our method	**49.67**	**44.07**	**0.9920**	**0.9772**

**Figure 4:**
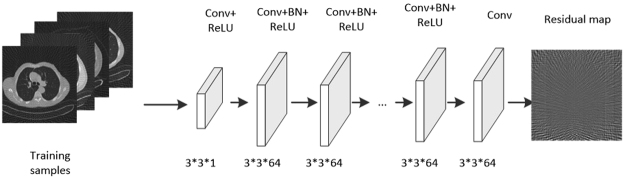
The flowchart of the AFCNN method.

Does the multi-scale improved GoogLeNet works better than one scale? We use the one scale feed-forward convolutional neural network to compare. The one scale CNN’s flowchart is shown in Fig. 4.

**Figure 5:**
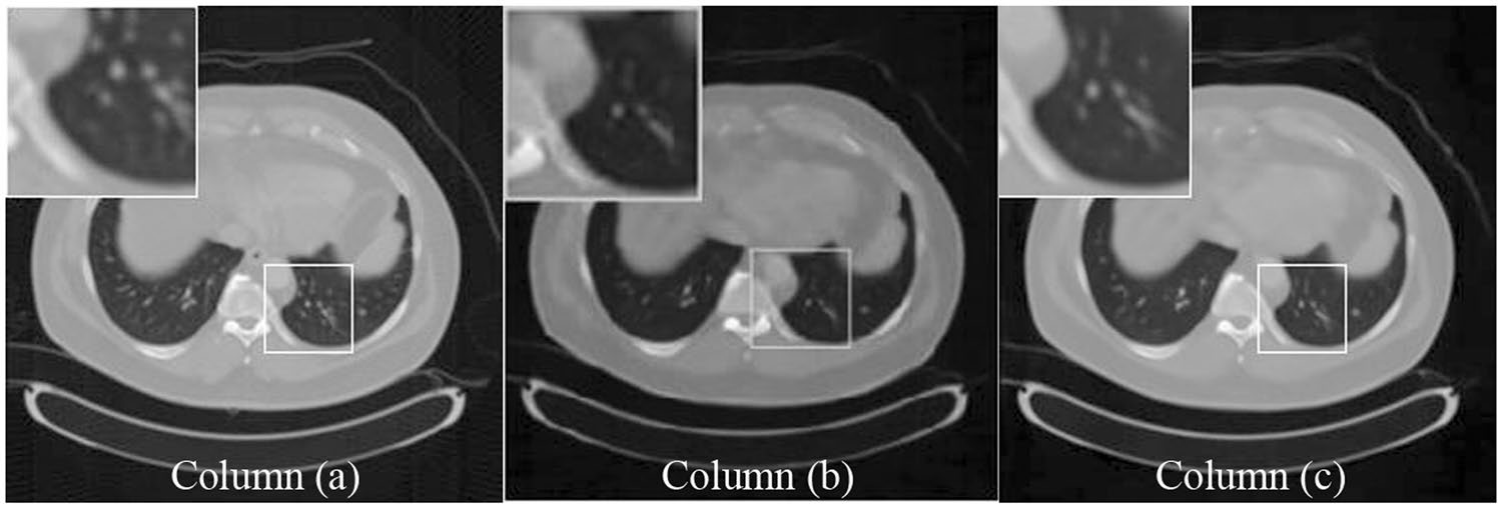
The image reconstructed by 60-views. Column (**a**) shows the reconstruction by the FBP method from the full projection views. Column (**b**) shows the reconstruction with artifacts by the one scale CNN method from the sparse projection views. Column (**c**) shows the artifacts-free reconstruction by the proposed method.

As shown in Fig. 5 and Table 6, we can clearly see the multi-scale improved GoogLeNet works better than the one scale CNN.

**Table 6:** Average values of PSNR and SSIM between proposed method and full-view FBP reconstruction for 256*256 CT images.

	120-views PSNR	60-views PSNR	120-views SSIM	60-views SSIM
One scale CNN	48.61	42.44	0.9905	0.9662
Multi-scale CNN	**49.67**	**44.07**	**0.9920**	**0.9772**

In this paper, we use improved GoogLeNet for the artifacts learning. The sparse-view reconstructed images *f* is much more like the full view reconstructed images than the artifacts *n*(especially when the artifacts level is low). Thus, typical mapping function *F*(*f*) would be closer to an identity mapping than CNN’s mapping function *μ*(*f*), and the artifacts learning for improved GoogLeNet is more suitable for remove artifacts.

We develop the GoogLeNet model and use it that outperforms the FBP method, ADS-POCS method and one scale CNN model. FBP method is the classical method. It has been used in the clinical CT system. But in the urtal-sparse view reconstruction, the artifacts were severe and covered all the information using FBP method. Although ASD-POCS preserved some structures, the details were heavily blurred and it is high computational complexity. As shown in Figs 1 and 2, our network preserved the details better than ASD-POCS. It also has the quickly computation speed. The intensity of reconstruction using the proposed method is very close to the full-view projective image.

The proposed method approaches to the gold standard image. The results indicate that the improved GoogLeNet algorithm is practical and effective for reducing the streak artifacts caused by projection missing in sparse CT reconstruction and preserving the quality of the sparse-view reconstruction CT image.

## Methods

Recently, the CNN has shown great success in handling various tasks. This work focuses on the design and learning of CNN for de-artifacts of the sparse-view CT reconstruction.

**Figure 6:**
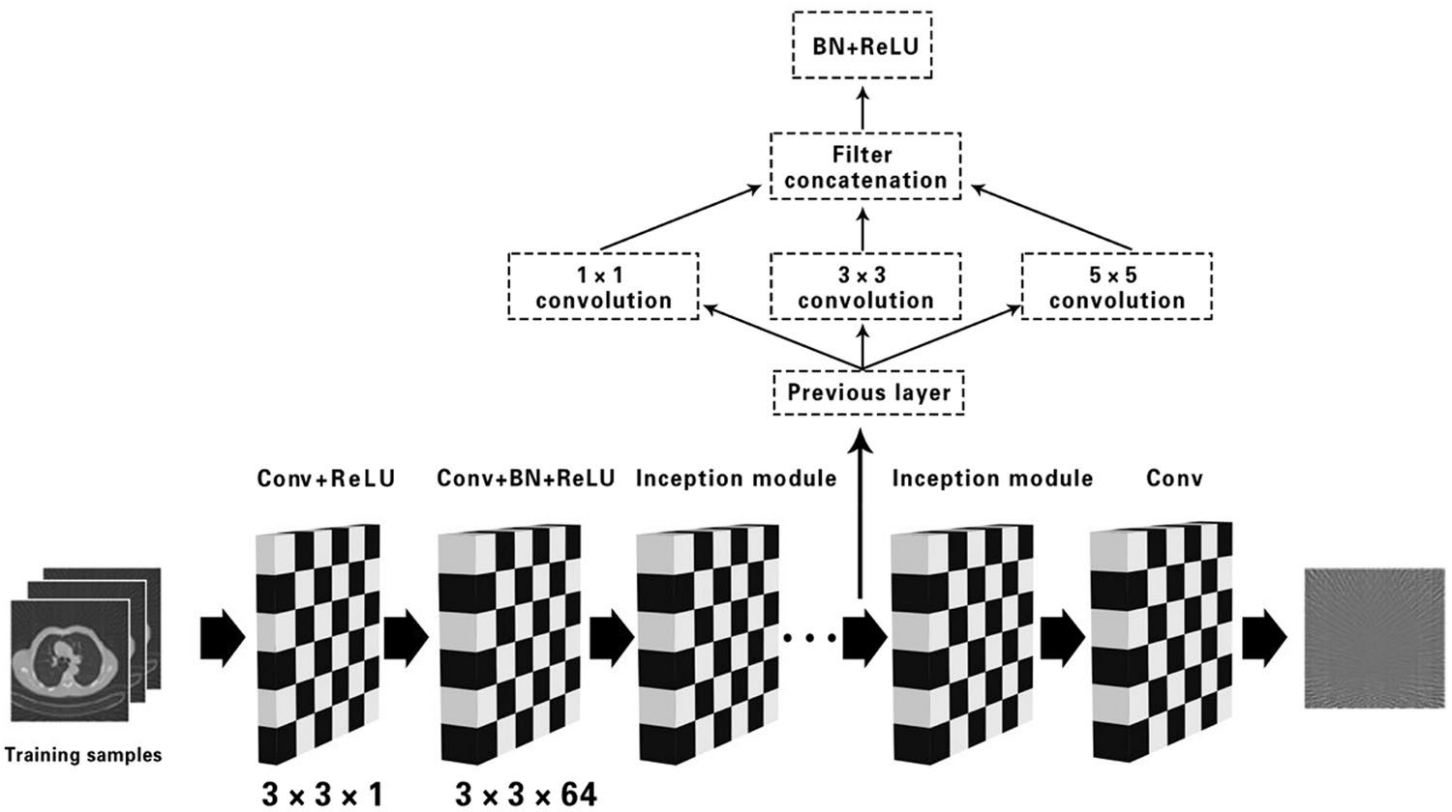
The flowchart of the proposed method.

In this paper, we apply a GoogLeNet^26^ (GN) based post-processing approach to remove the artifacts in the sparse-view CT reconstruction. GoogleNet is a deep convolutional neural network architecture that achieves the new state of the art for classification and detection. The most important in GN is the inception modules, which has the multiscale convolutional kernels. We restricted the current incarnations of the inception architecture with different filter sizes and showed it in Fig. 6.

This paper uses the residual learning for Improved GN to study the artifacts of sparse-view CT reconstruction, and then subtracts the artifacts which obtained by learning from the sparse reconstructed images, finally recovers a clear correction image.

The model of sparse-view reconstructed image *f* = *x* + *n*, which include the artifacts *n*, is similar to the model of image with noise. A mapping function *F*(*f*) = *x* is learned to predict the clear image in the typical de-noising models such as MLP^10^. In this paper, we use the Improved GN to train a mapping function *μ*(*f*) ≈ *n* and then use it to get the residual image *n*. The averaged mean squared error between the artifacts which is the difference of reconstructed images and the artifacts of network training is used as the loss function to measure the recovery image, and the formula is

1 $$l({\rm{\Theta }})=\frac{1}{2N}\sum _{i=1}^{N}||\mu ({f}_{i};{\rm{\Theta }})-({f}_{i}-{x}_{i})|{|}^{2},$$

which ${\{({f}_{i},{x}_{i})\}}_{i=1}^{N}$ represents N pairs of images it contains artifacts and real images, *μ*(*f*) is the CNN’ mapping function. In order to ensure the quality of the recovery image, it is necessary to train the parameters Θ in the network to obtain the appropriate parameter values so that the mean square error is minimized.

**Figure 7:**
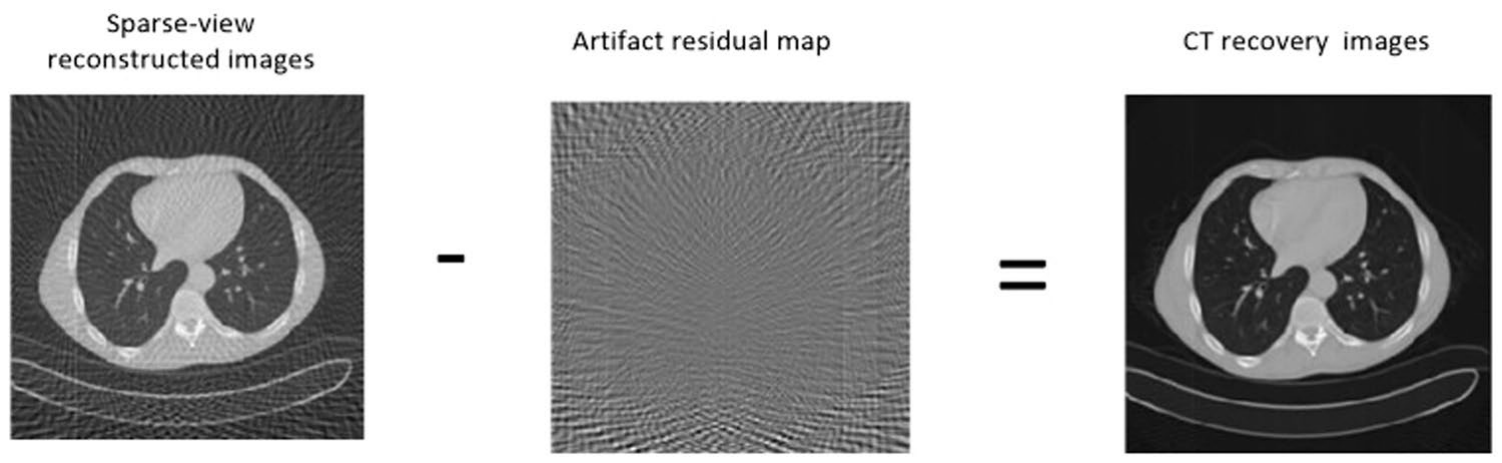
The process of removing artifacts from sparse reconstructed images in CT.

The artifacts image is obtained from the Improved GN, and then the final clear recovery image is obtained according to the algorithm *x* = *f* − *n*, which is shown in Fig. 7.
